# Supplementary material for: A suite of PCR-LwCas13a assays for detection and genotyping of Treponema pallidum in clinical samples
Source: Nat Commun. 2022 Aug 9;13:4671. doi: 10.1038/s41467-022-32250-y (PMC9362966; doi:10.1038/s41467-022-32250-y)
Supplement: Supplementary file 1 — Supplementary Information [file 41467_2022_32250_MOESM1_ESM.pdf]

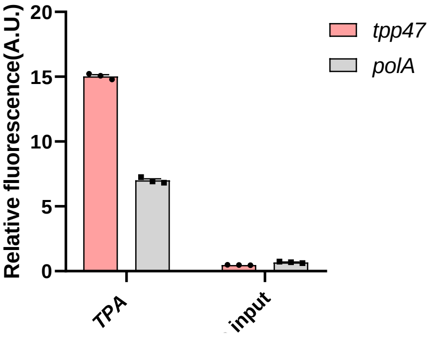

Figure S1. Fluorescence measurement of *tpp47* and *polA* for *Treponema pallidum* DNA. The PCR-LwCas13a assay produces a higher fluorescence value for *tpp47* than for *polA* after 180 min. n = 3 technical replicates; error bars represent mean ± SEM. Abbreviations: A.U., Arbitrary units. Source data are provided as a Source Data file.

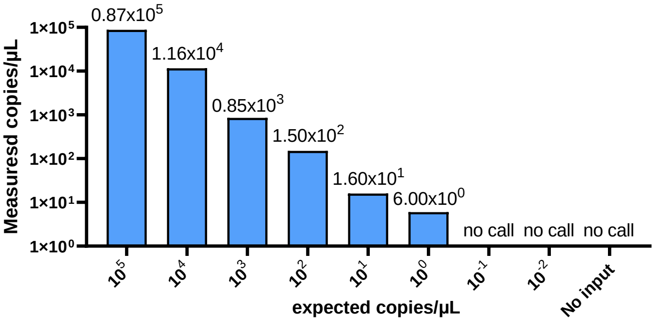

Figure S2. Quantification of *tpp47* dsDNA template by droplet digital PCR (ddPCR). Serially diluted aliquots of the *tpp47* dsDNA template were quantified by ddPCR. The X-axis represents expected concentration, and the Y-axis represents measured concentration. The measured value is labeled above the bar chart. Source data are provided as a Source Data file.

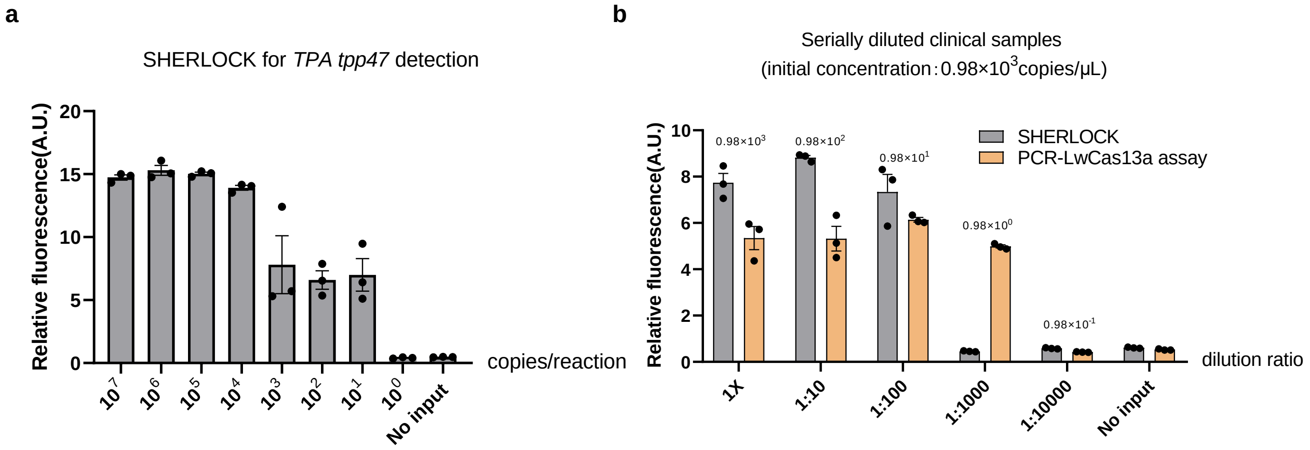

Figure S3. Performance of SHERLOCK for *TPA tpp47* detection. a SHERLOCK exhibits a limit of detection of 10 copies/reaction for *TPA tpp47*. **b** Head-to-head comparison of SHERLOCK and the PCR-LwCas13a assay using serially diluted clinical samples. n = 3 technical replicates; error bars represent mean ± SEM. Abbreviations: A.U., Arbitrary units. Source data are provided as a Source Data file.

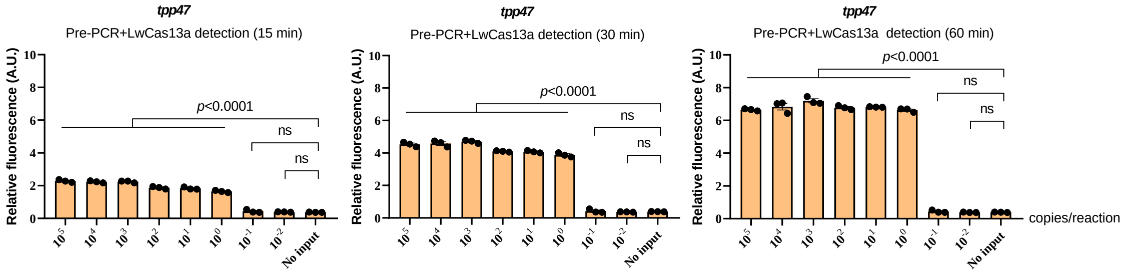

Figure S4. Fluorescence measurement of *tpp47* dsDNA detection using different LwCas13a detection incubation times. Series diluted aliquots of the *tpp47* dsDNA template were pre-amplified by PCR, followed by 15 min, 30 min, and 60 min LwCas13a detection. n = 3 technical replicates; error bars represent mean ± SEM. Two-tailed Student t-test was used to analyze the statistical significance; *p* value was labeled on figures, ns = not significant. Abbreviations: A.U., Arbitrary units. Source data are provided as a Source Data file.

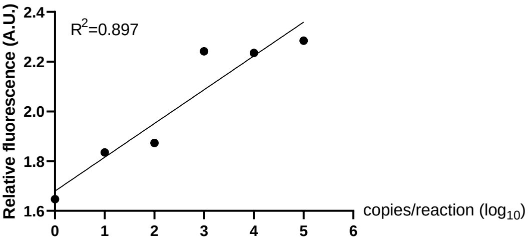

Figure S5. Correlation of copies number of *tpp47* dsDNA with detected fluorescence. Serially diluted aliquots of *tpp47* dsDNA template were pre-amplified by PCR. Fluorescence signals were measured after 15 min of LwCas13a detection. n = 3 technical replicates; values represent mean. Abbreviations: A.U., Arbitrary units. Source data are provided as a Source Data file.

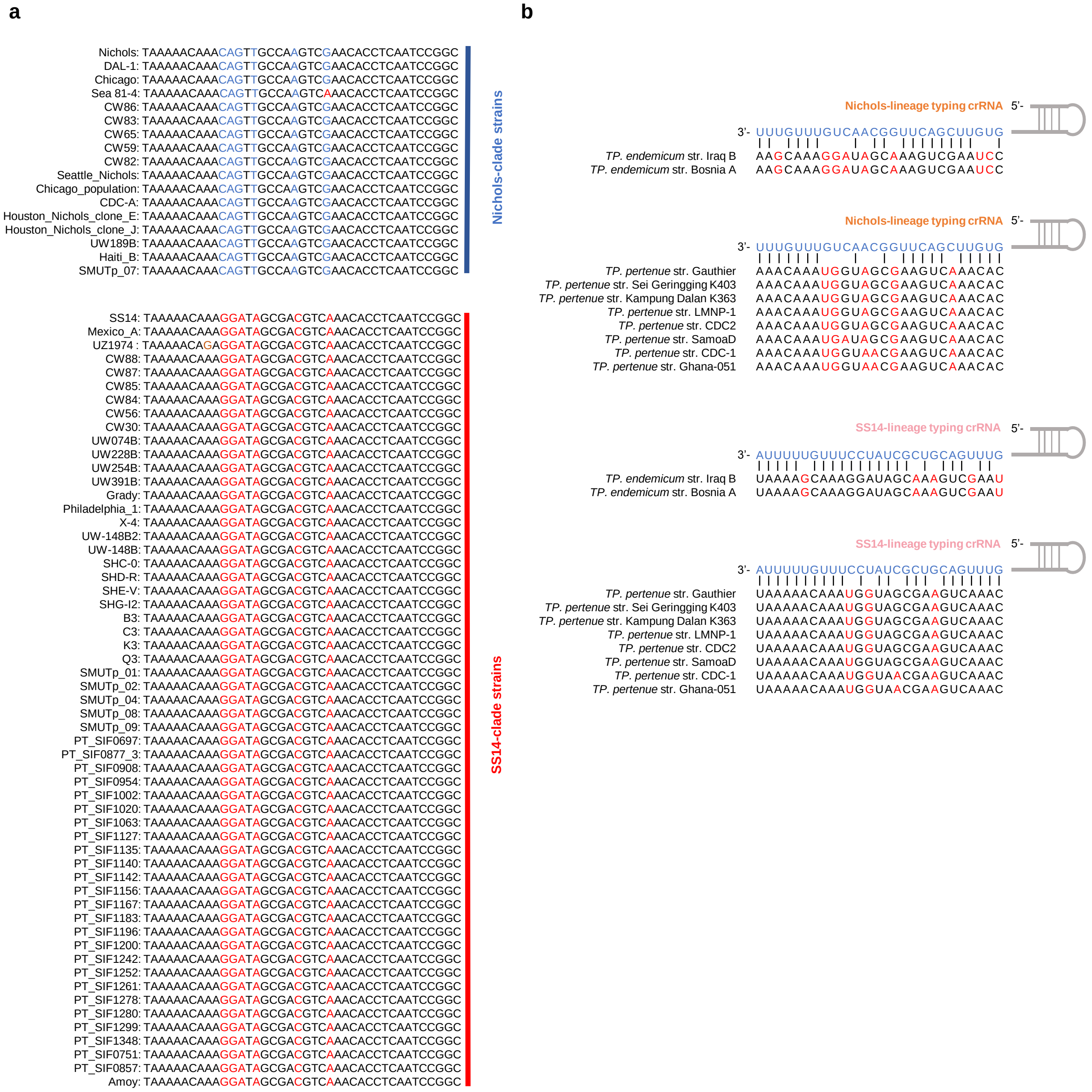

Figure S6. Target sequences of crRNAs used for determination of *Treponema pallidum subsp. pallidum* lineage. a The variable region of *tp0548* in Nichols- and SS14-clade strains was used to design crRNAs for lineage determination. **b** Compared to the closely related pathogenic treponemes *Treponema pallidum subsp. endemicum* (TEN) and *Treponema pallidum subsp. pertenue (TPE),* multiple mismatches were present in Nichols- and SS14-lineage typing crRNAs targeting the *tp0548* locus and are expected to yield *TPA* specificity.

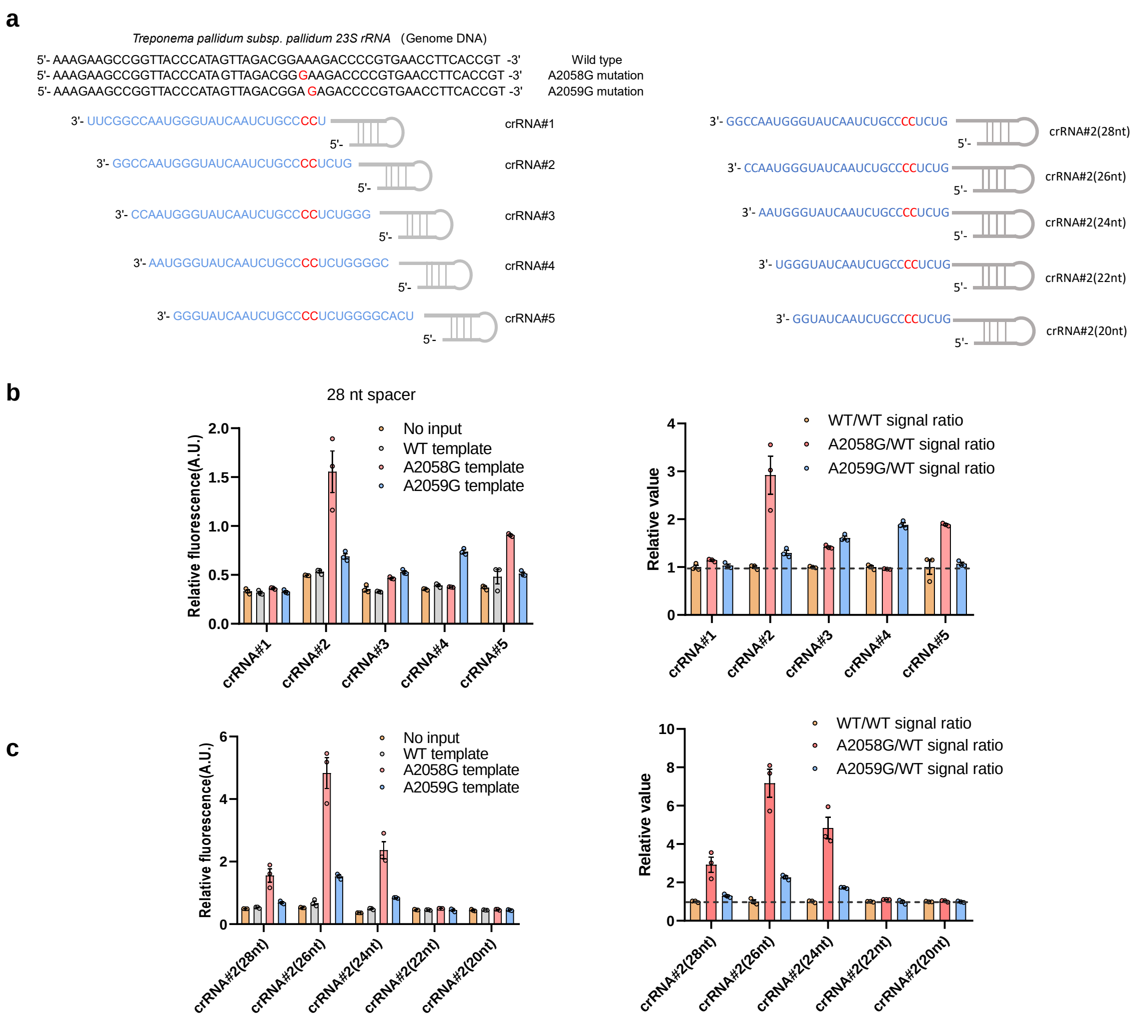

Figure S7. crRNAs screened for genotyping *Treponema pallidum 23S rRNAs*. a Synthetic crRNAs with mismatches used in this study. **b** Collateral cleavage activity on *23S rRNA* for 28 nt spacer crRNAs with different synthetic mismatches (labeled #1-5). Specificity ratios are calculated as the ratio of the on-target RNA (A2058G or A2059G mutation) collateral cleavage to the off-target RNA (wildtype). **c** Collateral cleavage activity on *23S rRNA* for crRNA#2 with different lengths (20-28 nt spacer crRNAs). Specificity ratios are calculated as the ratio of collateral cleavage of the on-target (A2058G or A2059G mutation) to off-target (wild-type) RNAs. n = 3 technical replicates; error bars represent mean ± SEM. Abbreviations: A.U., Arbitrary units. Source data are provided as a Source Data file.
